# Supplementary material for: High-performance reconstruction of microscopic force fields from Brownian trajectories
Source: Nat Commun. 2018 Dec 4;9:5166. doi: 10.1038/s41467-018-07437-x (PMC6279749; doi:10.1038/s41467-018-07437-x)
Supplement: Supplementary file 1 — Supplementary Info [file 41467_2018_7437_MOESM1_ESM.pdf]

High-Performance Reconstruction of Microscopic Force Fields from Brownian Trajectories

Pérez García *et al.*

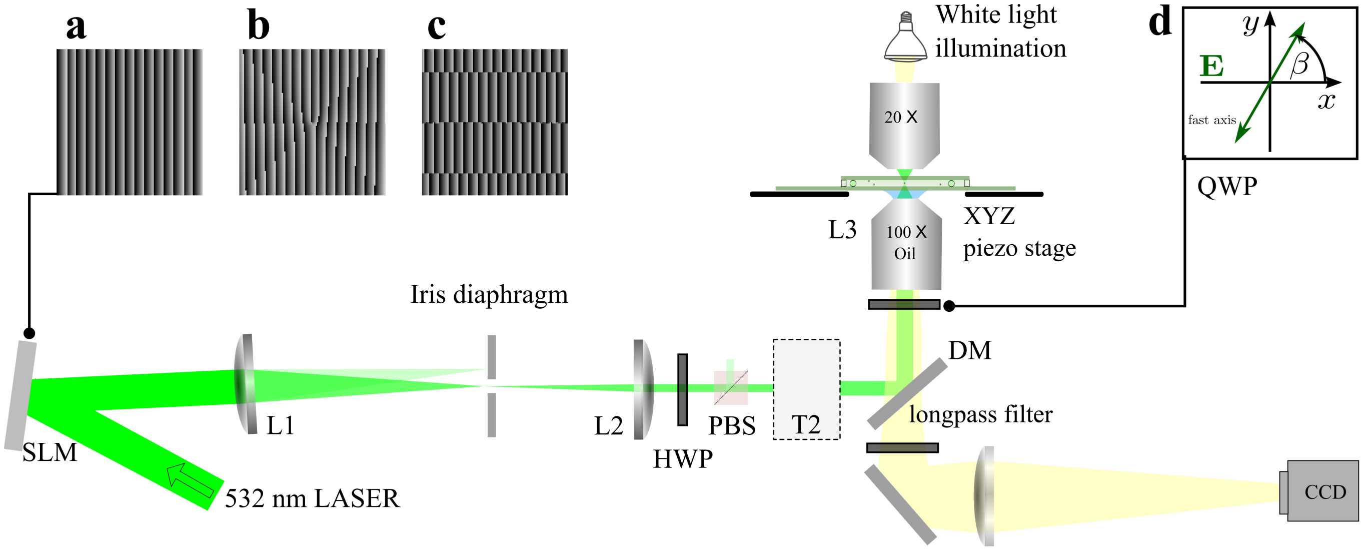

Supplementary Figure 1. **Holographic optical tweezers setup.** An expanded laser beam (wavelenght 532 nm) is reflected by a spatial light modulator (SLM, Hamamatsu X10468-04), through a telescope (lens L1, f = 400 mm, and lens L2, f = 175 mm), where the diffracted field of interest is discriminated by the iris diaphragm, a half-wave plate (HWP), a polarising beam splitter (PBS), and a second telescope (T2, magnification $M = 3$); it is then reflected by a dichroic mirror (DM), and finally goes through a quarter wave plate (QWP) to be focused by an objective (Carl Zeiss, 100×, oil immersion, 1.30 NA) within the sample chamber. The trapped particle is visualised through an imaging system consisting of a diode-based white-light illumination, a condenser (20×, 0.75 NA), the 100× objective, a tube lens (f = 200 mm), and a CMOS Camera (Basler acA800-510um with a ROI of 256 × 40 pixels and exposure time of 100 μs in the acquisition configuration). The shape of the beam is controlled by means of the SLM: (**a**) blazed grating phase modulation used to generate a single optical tweezers (Fig. 2); (**b**) blazed grating plus a $l = 1$ azimuthal phase to generate a LG_1 beam (Fig. 3); (**c**) two identical tweezers with a phase shift π to generate a multiwell potential (Fig. 4). (**d**) The QWP permits us to switch the polarisation state of the beam between linearly polarised ($\beta = 0$, Figs. 3d and 3g), circularly (+) polarised ($\beta = +\pi/4$, Figs. 3e and 3h), and circularly (−) polarised ($\beta = -\pi/4$, Figs. 3f and 3i).

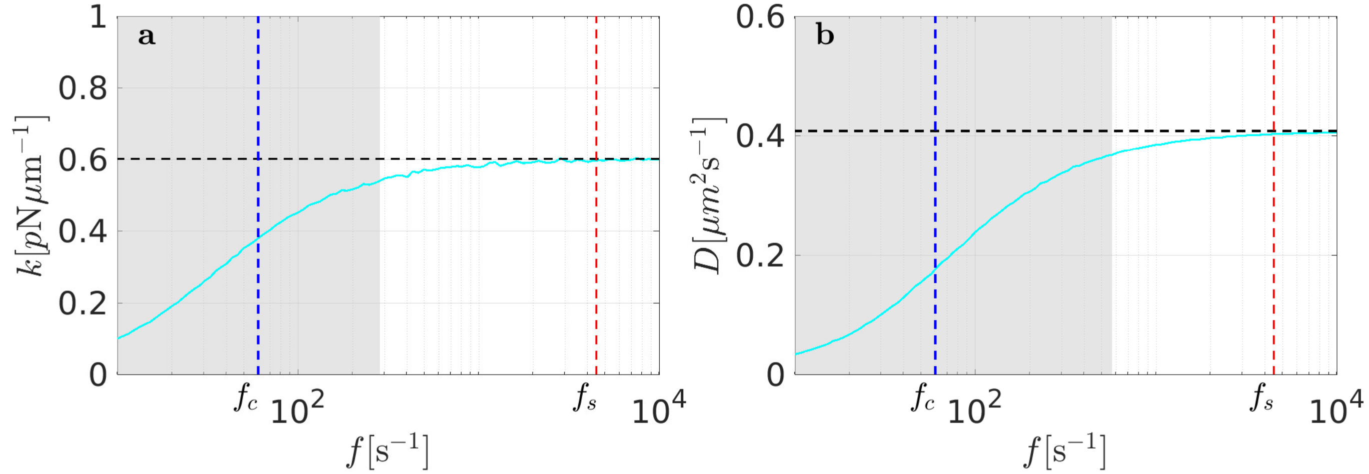

Supplementary Figure 2. **Performance of FORMA as a function of sampling time.** Values obtained by FORMA from simulated timeseries of an optically trapped particle for (**a**) the trap stiffness k and (**b**) the diffusion coefficient D as function of the sampling frequency f. The vertical blue and red lines respectively indicate the characteristic frequency in the trap ($f_c = k/\gamma = 60.3\,\mathrm{s}^{-1}$) and the sampling frequency ($f_s = 4504.5\,\mathrm{s}^{-1}$) of the results reported in Figure 2. The shaded areas represent the regions where the error in the determinations is greater than 10%. In all cases, we have simulated 24 trajectories of the motion of a spherical microparticle with radius $R = 0.5\,\mu$m in an aqueous medium of viscosity $\eta = 0.0011\,\mathrm{Pa\,s}$. The time for each simulations is fixed to 22 s.

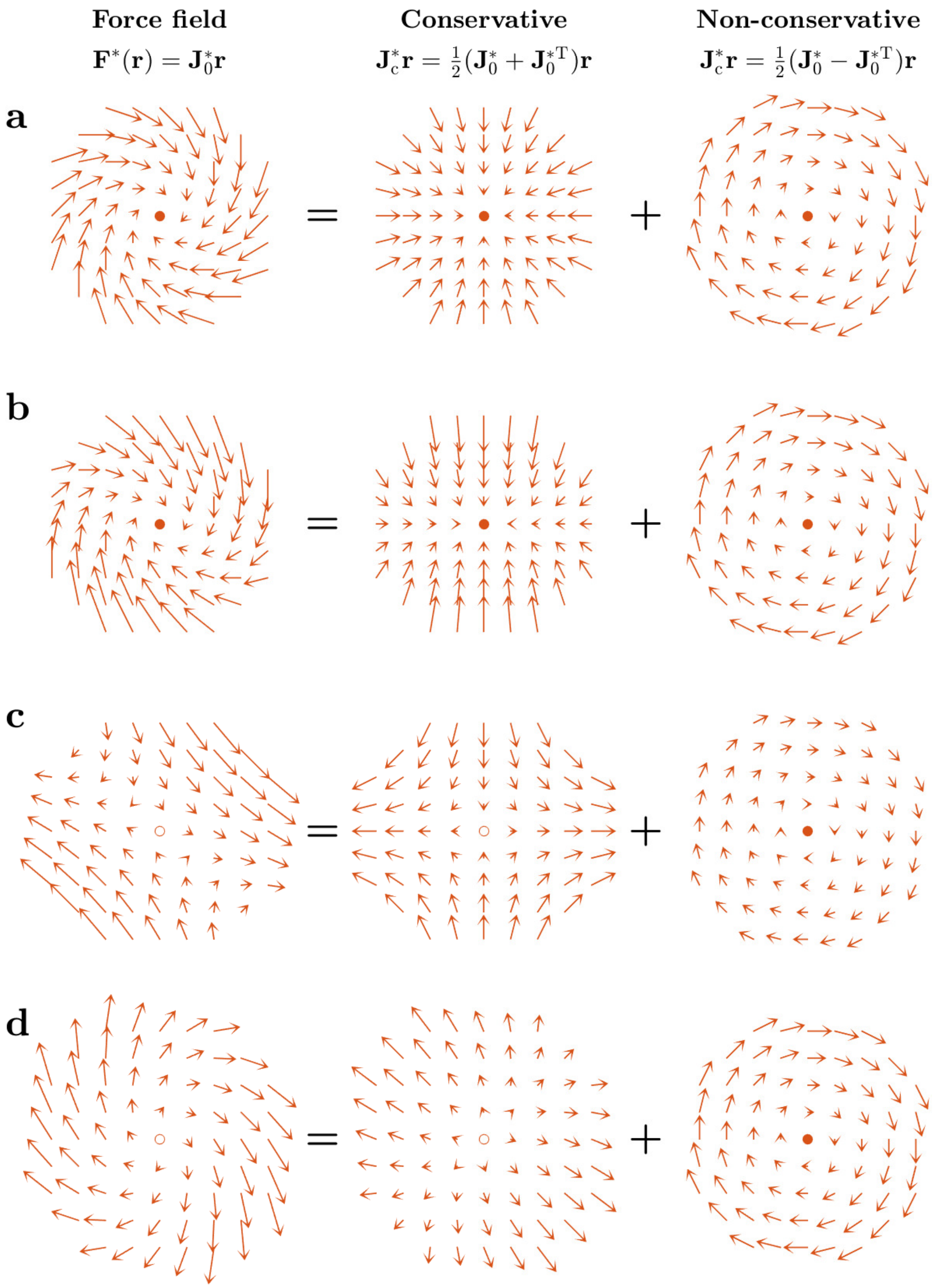

Supplementary Figure 3. **Examples of force fields.** Examples of force fields and their decomposition into conservative and non-conservative components (**a**) Isotropic harmonic potential where $k_1 = k_2 > 0$. (**b**) Elliptical harmonic potential where $0 < k_1 < k_2$. (**c**) Saddle point where $k_1 < 0$ and $k_2 > 0$. (**d**) Unstable point where $k_1 < k_2 < 0$ and the axes are titled by $\theta = 45^\circ$. The full and empty circles indicate the stable and unstable equilibrium points respectively.

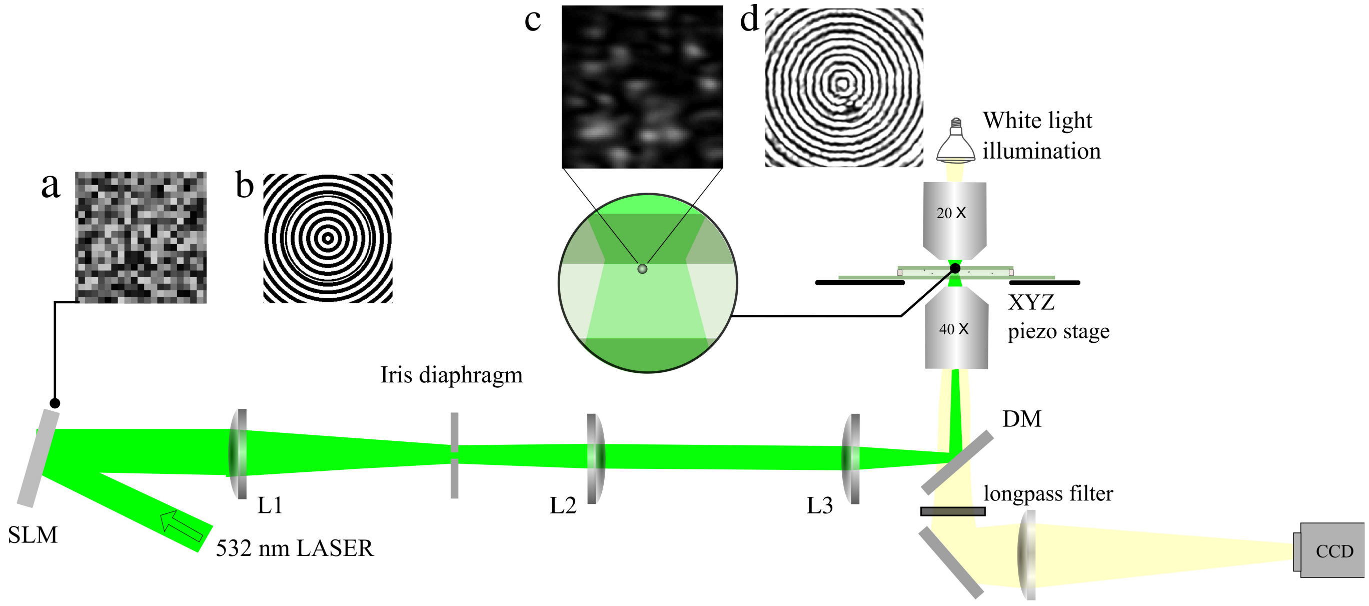

Supplementary Figure 4. **Speckle optical tweezers setup.** Optical micromanipulation with an image speckle. An expanded laser beam (wavelength 532 nm) is reflected by the spatial light modulator (SLM, Hamamatsu X10468-04), goes through a telescope (lens L1, $f = 400$ mm, and lens L2, $f = 100$ mm) where it is spatially filtered by an on-axis iris diaphragm (aperture $D_\mathrm{i} = 1.54$ mm), passes through a dichroic mirror (DM), is focused by lens L3 ($f = 150$ mm) on the back-focal plane of an objective (Olympus, 40×, 0.65 NA). The particle is visualised through an imaging system similar to that described in Supplementary Figure 1. (**a**) A uniform-distributed random phase mask is projected on the SLM to generate the speckle pattern (**c**), and (**b**) a ring-like dynamic phase distribution is used to set the initial position of the particle within the area of interest (**d**). Once the particle is in the desired initial position, the SLM projects the random phase distribution to generate the speckle pattern, and the particle position is recorded as it diffuses for about 6 seconds, then the speckle pattern is changed for the ring-like trap to set the initial position, this process is repeated 500 times. The polystyrene particle is trapped in 2D as it is pushed towards the upper coverslip by the beam radiation pressure.

coord.	k (pN μm^{-1})	k^* (pN μm^{-1})	Ω (s^{-1})	Ω^* (s^{-1})	D (μm^2 s^{-1})	D^* (μm^2 s^{-1})
x	0.6	0.60 ± 0.02	2	2.27 ± 1.15	0.392	0.387 ± 0.001
y	0.5	0.49 ± 0.02	1.5	1.57 ± 0.81	0.392	0.387 ± 0.002
z	0.2	0.20 ± 0.01	3	2.86 ± 0.78	0.392	0.389 ± 0.001

Supplementary Table 1. **Estimated parameters in a non-conservative 3D trap using FORMA.** Results of the analysis with FORMA of a 3D Brownian simulation, assuming a harmonic trap with different k's and Ω's along the three coordinate axes. We simulated a spherical particle with radius $R = 0.5\,\mu$m in an aqueous medium of viscosity $\eta = 0.0011$ Pa s at a sampling frequency $f_s = 4504.5$ s, and we used 24 windows of $2 \cdot 10^5$ samples for the averaging.

Eq. point	x^*_{eq} (μm)	y^*_{eq} (μm)	k^*_1 (pN μm^{-1})	k^*_2 (pN μm^{-1})	θ^* (deg)	Ω^* (s^{-1})	N_{s}
1	1.79	8.08	0.14	0.07	358	0.47	7486
2	4.41	7.66	0.21	0.04	37	−0.07	175406
3	1.41	7.38	0.20	−0.09	15	0.67	830
4	2.11	7.08	0.03	−0.08	116	−0.69	562
5	5.61	6.72	0.11	0.07	105	−4.64	1240
6	5.21	6.58	0.04	−0.10	108	1.58	1084
7	1.91	6.58	0.04	−0.11	63	−0.14	841
8	0.75	6.22	0.10	0.08	56	−0.25	41667
9	4.71	6.08	0.06	0.00	358	1.24	13144
10	3.11	6.02	0.07	0.01	49	−1.43	5411
11	3.81	5.98	−0.05	−0.06	32	−4.87	2101
12	5.61	5.78	0.07	−0.05	124	0.59	626
13	1.51	5.78	0.03	−0.12	43	1.09	1146
14	3.21	5.58	−0.01	−0.05	12	2.10	2369
15	3.61	5.38	−0.03	−0.07	11	−2.82	1829
16	6.27	5.12	0.21	0.18	113	−0.00	123870
17	4.85	5.08	0.02	−0.04	3	0.17	6154
18	1.11	5.08	0.08	0.03	352	0.10	1340
19	2.27	4.74	0.16	0.14	5	−0.07	486547
20	3.43	4.60	0.07	−0.03	89	−0.86	4819
21	5.43	4.44	0.02	−0.09	126	3.14	5379
22	4.71	4.38	0.08	0.00	99	−0.32	26013
23	1.41	4.38	0.11	−0.06	62	0.55	14948
24	3.51	4.28	−0.03	−0.04	339	0.89	3920
25	1.05	4.20	0.12	0.06	94	−0.53	32006
26	6.01	3.98	0.02	−0.06	53	0.14	1188
27	3.95	3.98	0.02	−0.12	330	−0.58	4590
28	5.27	3.96	0.08	0.04	125	−0.74	17540
29	2.61	3.78	0.04	−0.13	35	−2.89	2561
30	2.41	3.48	−0.01	−0.11	55	−0.07	3546
31	5.67	3.42	0.05	−0.04	332	−0.80	3564
32	1.11	3.38	−0.03	−0.08	17	−2.75	1220
33	4.31	3.34	−0.03	−0.08	356	−2.03	2505
34	7.23	3.32	0.22	0.14	27	−0.30	42399
35	3.19	3.24	0.10	0.05	73	−0.17	100763
36	2.31	2.98	0.07	−0.05	78	0.18	3440
37	5.07	2.92	0.13	−0.02	80	−3.78	1729
38	5.91	2.88	0.02	−0.04	71	4.53	1411
39	4.81	2.68	−0.00	−0.10	352	1.58	1407
40	5.71	2.58	0.03	−0.07	121	2.57	1449
41	3.71	2.48	0.05	−0.01	356	−1.62	7907
42	1.45	2.44	0.14	0.13	21	−0.64	57803
43	3.01	2.08	0.05	−0.10	106	−4.62	1529
44	1.01	0.76	0.16	0.07	76	−0.27	18292
45	4.69	0.72	0.10	−0.03	105	−0.22	16629
46	4.05	0.72	0.09	0.02	39	0.21	34384
47	5.15	0.56	0.14	0.06	111	−0.49	27232

Supplementary Table 2. **Equilibrium points identified by FORMA in a speckle parttern.** For each equilibrium point identified in the data shown in Figure 5, FORMA provides the position $(x^*_{\mathrm{eq}}, y^*_{\mathrm{eq}})$ (from the lower left corner of Figure 5a), its stiffnesses k^*_1 and k^*_2 along the principle axes, the orientation θ^* of the principle axes with respect to the Cartesian axes, and the frequency Ω^* associated to the non-conservative (rotational) component of the force field. N_{s} is the number of measurements of the particle displacements used by FORMA for the estimation.
